# The Medical Ideal

**Published:** 1907-10

**Authors:** 


### THE MEDICAL IDEAL.

In no profession or business is the influence of ideals of so much importance as in the practice of medicine. Self-interest is the chief guiding motive in ordinary business. That this must be cast aside to a great extent by the physician is obcious when one considers his opportunities of promoting his own interest in many ways that might not be in the least wrong if viewed from the commercial ideal, for the ethics of trade are not sufficient to secure honesty in the practice of medicine.

In his presidential address, Dr. A. Jeffries Turner* takes up the subject of the Medical Ideal and shows the necessity for higher standards in the physician than in the tradesman or those whose merchandise or services are of more evident or easily ascertainable value.

He says that if he had to classify the men he knows he would do so according to their ideals. Not that it would be an easy or a practicable standard, for a man with low ideals may give them free expression, but as a rule, there are few things about which men are more reticent. It requires an intimate acquaintance or a prolonged course of observation to form an estimate of the inward pattern which is continually influencing either consciously or subconsciously, the visible behavior.

That the true medical ideal means more than mere honesty and necessitates effacement of self interest is shown by the following instances: There are a few patients, mostly women, who are anxious to be relieved of some comparatively trivial or perhaps wholly imaginary trouble by surgical operation. They have no conception of the possible dangers or deleterious after effects of what they ask. Nothing else will please them. If the surgeon consult his own interest he will of course operate in these cases. The patient almost certainly recovers from her operation; she thinks she is better for a time and advertises the operator among her friends. Conversely, there are, of course, cases where immediate operation is to the best interest of the patient, but he is averse to it. Perhaps it is a case of sudden illness, for instance perforative peritonitis. The patient will very probably die after an operation and the friends will say the surgeon killed him. If by good hap he recovers they will not realize the imminent danger from which he has been saved. Here the temptation is to temporize, to please the patient and his friends by delay, to operate perhaps not at the best time for the patient but at the least dangerous time

*Australasian Medical Gazette for the surgeon's reputation.

Dr. Turner cites a number of other instances, which might be spread out indefinitely, where the guidance of an ideal shall be imperative. We all know too well that the way is always open for a medical man to increase his income by disregarding the ideals of his profession, and as a man's attainments are confined within the boundaries of his ideals we should be proud of the standard set by our profession.

Let those who doubt the value of ideals be watchful lest they forfeit the respect of their fellow-practitioners.

